# Medical Community Perspectives Regarding the Egyptian Medical Licensing Exam: A Mixed-Method Study

**DOI:** 10.7759/cureus.14636

**Published:** 2021-04-22

**Authors:** Asmaa Abdel Nasser, Asmaa F Sharif, Fatma Alzahraa A Elkhamisy, Hadeer Adel, Ahmed Hussein, Nesrin M Handoka, Amira Farghaly, Ahmed K Ali, Enjy Abouzeid

**Affiliations:** 1 Medical Education Department, Faculty of Medicine, Suez Canal University, Ismailia, EGY; 2 Medical Education Unit, Ibn Sina National College, Jeddah, SAU; 3 Forensic Medicine and Clinical Toxicology Department, Tanta University, Tanta, EGY; 4 Clinical Medical Sciences Department, College of Medicine, Dar Al Uloom University, Riyadh, SAU; 5 Pathology Department, Helwan University, Cairo, EGY; 6 Pediatrics Department, Armed Forces College of Medicine, Cairo, EGY; 7 Pediatrics Department, Port Said University, Port Said, EGY; 8 Medical Education Department, Suez Canal University, Ismailia, EGY; 9 Medical Education Unit, College of Medicine, Prince Sattam Bin Abdulaziz University, Al Kharj, SAU; 10 Medical Education Unit, American University of Beirut, Beirut, LBN

**Keywords:** exam set up, national licensing exam, egypt, emle framework, exam logistics

## Abstract

Background: Although national licensing examinations (NLEs) may be a costly process, they can predict performance of medical practitioners for many years following graduation. The current licensing requirements do not fulfill this function as there are no clear performance criteria for them. Therefore, new requirements should be developed and announced.

Objective: The study aims to develop a framework for the Egyptian Medical Licensing Exam (EMLE) by exploring the opinions and perceptions of Egyptian health practitioners and medical educators.

Methods: This study is a two-phase exploratory mixed-method study. An online discussion forum was conducted with medical practitioners and educators concerning the development of the EMLE. Then, an online survey was distributed to explore the opinions of medical practitioners and educators about the EMLE.

Results: Fifty medical practitioners and educators participated in the discussion forum about the development of the EMLE, while 266 participants responded to the online survey. The responses of the participants contributed to the development of a framework for the EMLE that is divided into two main sections, the exam logistics and the exam set up. The exam logistics included the exam committee, prerequisites for the exam, the admission criteria and fees, and validity of the license. The exam set up included exam setting, structure, pass marks, and exam retake policy.

Conclusion: The study concluded that medical practitioners and educators could contribute greatly to the planning for the EMLE. Their opinions are based on their experiences and include the timing of the exam, blueprinting, assessment methods, psychometrics and retake.

## Introduction

In response to the increased demand for social accountability over the last 20 years, many countries have shown a greater tendency towards improving medical regulations. This in turn created the need for extensive examinations to assure that minimum standards for competent medical graduates are being met [1]. Furthermore, there has been increased mobility of medical professionals in recent years, in addition to the dependence of some countries on international medical graduates [2]. It has been recorded that many professionals cross borders to get a chance to become physicians in more lenient settings [3]. All these factors highlight the importance to adapt licensing procedures to ensure professionals’ quality, patient safety, and public trust maintenance. One of the main methods to ensure healthcare providers’ quality, adherence to regulations, and meeting optimum care standards is to apply licensing procedures on all future physicians. Although this may be costly, there is evidence that supports the ability of licensing exams to predict better performance of healthcare professionals for many following years. National licensing exams (NLEs) could screen doctors with poor performance and improve their competencies, consequently enhancing medical education standards, quality, and patient safety [3].

Many economic, political, and socio-demographic factors affect the regulation and licensing of novice doctors, causing variation from one country to another [4]. The available literature captured four essential different approaches to licensing examinations that range from an approach where all prospective doctors are required to pass the exam to an approach where no NLEs were in operation [2]. That being said, there are common criteria that are reported for the majority of licensing exams. These include: (i) meeting educational requirements from a nationally accredited medical school, (ii) completing a supervised and authorized clinical training period, (iii) ensuring candidates’ competency, and finally (iv) passing NLEs [3].

In Egypt, the Egyptian Medical Syndicate (EMS) and the Ministry of Health and Population (MOHP) regulate the registration and licensing of physicians [5]. Until recently, medical students who successfully completed six years of medical education and one year as house officers are granted the Bachelor degree of Medicine and Surgery (M.B.B.Ch). This allows the graduates to register, obtain their license, and become general practitioners [6]. There is no NLE in operation despite a large number of medical schools, different curriculum approaches, and different instructional and assessment methods applied. However, the absence of an NLE in Egypt is compensated by the implementation of accreditation schemes to ensure a high quality of education. Still, the accreditation standards are not obligatory. Even if they are encouraged, many medical schools are still in the process of being accredited [7].

There are many challenges that impact the quality of medical education in Egypt. For instance, there is an increasing need to improve infrastructure capacities and financial resources allocated for medical schools [8-10]. In addition, there is a yearly increase in the number of Egyptian medical students, which implicates class density, interaction with teachers, and their peers [11]. Although there is a high number of registered medical practitioners, many of them work in different countries resulting in the continuous insufficiency of well-trained physicians in Egypt, which creates a vicious cycle [12]. This cycle aggregates with increased needs to improve medical education quality, ensure practitioners’ competency, and meet the demands of establishing new medical schools. Currently, Egypt has 29 undergraduate medical education programs, with a sharp increase in private schools in the past few years [13]. Thus, reforming medical education in Egypt is currently being done by authorities without the usage of any kind of assessment. This reform focuses on unifying outcomes, using integrated curricula, early clinical exposure, and adopting innovative teaching and assessment methods [6]. Recently in Egypt, the Supreme Council of Universities endorsed an undergraduate medical education curriculum named "5+2" that changes the duration of study in medical schools to five years (two preclinical + three clinical) based on a credit hours/points system. These five years are followed by two years of supervised clinical practice followed by a national medical licensing exam [14].

A proposal for the Egyptian Medical Licensing Exam (EMLE) came to light in 2019. It was to be implemented in 2020 as a pilot for all graduates of medical schools across Egypt, but implementation was delayed due to the COVID-19 pandemic. The application of the Egyptian licensing exam is now supposed to be issued by the year 2023, with the graduation of the first batch of the new medical education curriculum (5+2). It would be helpful to learn about how the Egyptian medical practitioners and educators could contribute to the planning of this exam in terms of structure, logistics, pre-requisites, timings, etc. Therefore, this study aims to assist in the development and implementation of this important exam and help other international health systems that are willing to implement a medical licensing exam, especially those of a similar educational strategy. Accordingly, the current study presents a framework for the EMLE based on a deep investigation of Egyptian medical practitioners' and educators’ vision regarding the exam format and criteria.

## Materials and methods

Study design

**Figure 1:**
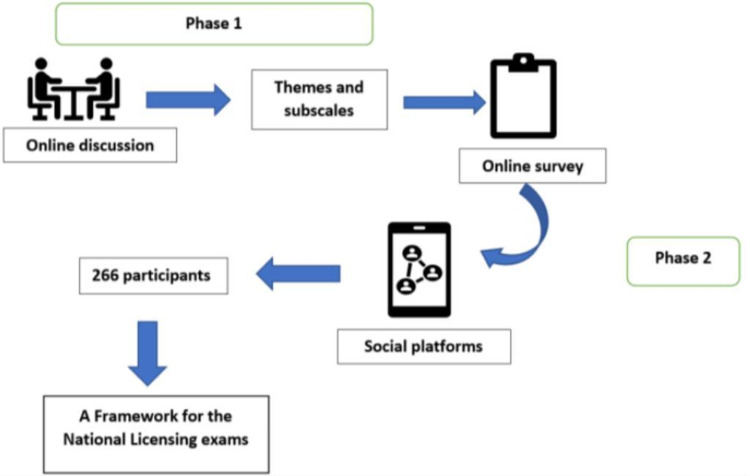
A Conceptual Model for the Study Phases

A two-phase exploratory mixed-method study was conducted and included both medical practitioners and educators as shown in Figure 1.

Phase one

Context (Design)

The first phase of the program was an online discussion forum held with Egyptian medical practitioners and medical educators. The forum was part of an online master’s program in medical education that includes students from different medical specialties, clinical and academic. The purpose of the discussions was twofold: (i), exploring the opinions of health professionals with respect to the purpose, context, preparation, and implementation of EMLE, in addition to (ii) designing a survey tool to explore the perceptions and opinions of medical practitioners and medical educators towards the EMLE.

Sampling

A voluntary response sample of 50 participants shared in the discussion forum. They were heterogeneous participants as they varied in gender, specialty, academic rank, working place and residence.

Tools and Data Collection

An online discussion (LISTSERV) was moderated by the researchers (Appendix 1). It was an asynchronous activity through email threads. The researchers prepared the discussion guide including the objectives, questions, case scenarios, and facilitators. The objectives of the discussion included exploring and critiquing participants’ opinions towards the EMLE regarding the criteria for development and admission, assessment methods, implementation, and certification/licensing requirements. The discussion was carried out over two weeks during February 2020. The discussion threads had reached 196 unique individual responses. The participants' responses were analyzed and summarized.

Analysis

The participants’ responses to the discussion were analyzed thematically, and in part, contributed to the design of the survey and its sections as this appears in their description in the second phase.

Phase two

Design

Based on the results of the analyzed responses of the online discussion, an online survey was developed. It aimed to explore the opinions on a wider scale of medical educators, academics, and practitioners regarding the EMLE. The survey was composed of 17 items within four sections: participants’ characteristics, EMLE admission criteria, the exam structure, and the medical license renewal process.

The survey was validated by 10 medical education experts from the Medical Education Department, Suez Canal University. The final version of the online survey was prepared and distributed, using Google forms (Appendix 2).

Data Collection

The survey was disseminated through various social media platforms to reach wide sectors of the medical community. The online mode was the most convenient method during the COVID-19 pandemic. These platforms included WhatsApp groups, Facebook groups and Facebook pages that gathered members from different medical practitioners’ and medical educators’ communities. Participants joined the study on a rolling basis over a month duration, March 2020. The survey was distributed on a wide scale among medical practitioners and medical educators in Egypt. A voluntary response sampling was applied in this study. It included 266 Egyptian medical educators and practitioners who responded to the online survey. The use of various social media platforms contributed to an equal chance for different Egyptian medical educators and practitioners to be included in the sample.

Statistical Analysis

The collected data were coded and analyzed using Statistical Package for Social Sciences (SPSS) version 27 (IBM Corp., Armonk, NY, USA). The results of the descriptive analysis were presented by calculating frequencies and percentages. Furthermore, bivariate analysis using a Pearson Chi-Square correlation test was used to compare differences in certain selected outcomes among groups of participants according to their affiliation and specialties. The results were considered statistically significant when p ≤ 0.05.

Ethical considerations

Ethical approval for the study was obtained from the Faculty of Medicine-Suez Canal University Research and Ethics Committee (FOMSCU 4317/2020). Data collection was done in accordance with the Helsinki Declaration [15]. All the participants were informed about the purpose of the study and their right to refuse participation or to withdraw from the study without consequences. The confidentiality of the responses was maintained as the questionnaire was provided anonymously.

## Results

Demographic data

Two hundred and sixty-six medical practitioners and educators responded to the survey. The study participants varied according to their professional rank as shown in Table 1. The study sample of both the survey and the discussion covered 17 Egyptian medical universities, 15 public and two private. Among the different specialties represented in the study sample, clinical educators and practitioners represented 69.4% of respondents, while academic educators represented 30.6%. A wide range of clinical disciplines was represented; 13 Internal Medicine subspecialties, 10 surgical subspecialties, and three basic science subspecialties.

**Table 1:** Characteristics of the Survey Participants (N = 266)

	n (%) *
Affiliation	
University staff	198 (74.4)
Ministry of Health staff	68 (25.6)
Specialty**	
Academic	78 (30.6)
Clinical	177 (69.4)
Rank	
Assistant, associate and full Professors	123 (46.2)
Lecturers, assistant lecturers, and demonstrators	75 (28.2)
Consultants	16 (6.0)
Specialists and commissioned doctors	52 (19.5)
Discrepancies in total number are attributed to missing values. * N=255	

What is the framework?

**Figure 2:**
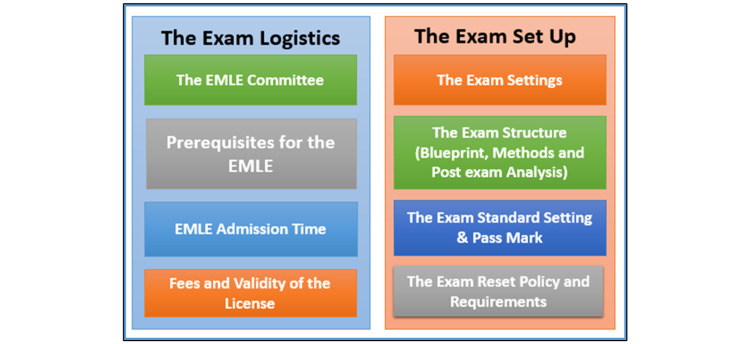
The Framework for the National Licensing Exam EMLE: Egyptian Medical Licensing Exam

The aim of the study was to develop a framework for the development of the EMLE exam through exploring the medical community’s opinions regarding the exam format and criteria. The input from the online discussion and the survey contributed to the development of the following framework that is divided into two main themes: A) The Exam Logistics and B) The Exam Set Up, as shown in Figure 2.

In the discussion forum, participants have highlighted the need for establishment of licensing exams for Egyptian medical graduates. A clinical educator stated, “from my point of view, establishment of EMLE is an urgent need, and it is applied on a wide scale in different countries worldwide”. An academic educator added “Application of this system serves different dimensions such as internationalization of the medical education, better opportunities for accreditation, and preparation of the medical graduates for the global world”.

Another clinical educator mentioned, “I think there are two important driving forces for the establishment of licensing exams; the increased number of medical schools in Egypt and the need for a standardized exam that ensures the achievement of common standards of medical knowledge and clinical skills among Egyptian graduates”. Another academic educator added “EMLE will ensure that standards are met and will maintain the high reputation of Egyptian physicians’.

A. The exam logistics

The EMLE Committee (A Multidisciplinary Team)

Participants in the discussion forum highlighted that EMLE should be conducted and maintained by several parties: representatives from staff members of medical schools and consulting experienced organizations in exam conduction. Also, they recommended the recruitment of medical education experts to assist in the development, administration and psychometric measures of these examinations in order to attain high test standards.

Also, the participants of the discussion provided suggestions regarding the committee for EMLE. They all agreed that the team should be multi-disciplinary and responsible about the planning, implementation, and evaluation of the exam. A clinical educator mentioned “The personnel in the multidisciplinary team should be clinical and basic science professors from different universities and ministry of health. Also, I recommended the presence of medical educationists, infection control, medical ethics and quality control specialists in the team”. While another academic educator added “I recommended that an independent subcommittee must revise and validate the difficulty of the questions included in the question bank prepared by the multidisciplinary team, and I suggested an intensive training should be provided for this team prior to the preparation of the question bank”.

Prerequisites For the EMLE 

Regarding the prerequisites of admission, all the participants agreed that the students should be holders of a bachelor degree and graduated from an accredited Egyptian - or international - medical school listed in the Egyptian Supreme Council of Universities. In addition, credit hours in social, behavioral and clinical competencies, were suggested. An English language certificate (TOFEL or IELTS) and computer skills courses were proposed as a must in the prerequisites. On the other hand, other respondents cited that a proof of attendance of Continuous Medical Education (CME) activities is also suggested.

According to the survey results, most of the participants selected the Basic Life Support (BLS) certificate (80.8%), an English language certificate (72.6%), a basic computer skills certificate (i.e. ICDL) (63.2%), and an accepted house officer’s portfolio/logbook (60.2%) as obligatory admission criteria for the EMLE (Table 2). There were some recommendations from respondents regarding the admission criteria that emphasized the importance of competence in emergency skills, and health education activities in the community.

**Table 2:** Survey Results “EMLE Logistics” (N = 266) EMLE: Egyptian Medical Licensing Exam

Items	n (%)
EMLE perquisites*	
Basic Life Support skills	215 (80.8)
English language skills	193 (72.6)
Computer skills (ICDL)	168 (63.2)
Qualified portfolio	160 (60.2)
Clinical course	99 (37.2)
EMLE Formative exam	87 (32.7)
Communication and presentation skills	65 (24.4)
Reference letter	63 (23.7)
Research competencies	56 (21.1)
Participation at education events (e.g. conferences)	44 (16.5)
Publications	27 (10.2)
Time of administration	
Directly after Internship	167 (63.0)
Within 2 months after internship	44 (16.2)
Within 3 months after internship	30 (11.3)
4 months or more	14 (5.3)
Others	11(4.2)
Fees	
Paid for each setting	30 (11.3)
Free for first setting only	174 (65.8)
Paid for promotion purpose only	46 (17.3)
Others	15 (5.6)
Validity and Expiry	
2 years	36 (13.5)
5 years	112 (42.1)
Promotion	104 (39.1)
Others	14 (5.3)
*Answers are not mutually exclusive	

As shown in Table 3, there is a significant difference between participants affiliated to universities and those working at Ministry of Health with respect to their choices of EMLE prerequisites, especially in the choice of house officer’s portfolio and reference letters. Furthermore, there was a significant difference between clinical and academic participants in regard to requiring clinical rotations/certificates as an EMLE admission criteria.

**Table 3:** “EMLE Logistics” by participants’ affiliation and specialty EMLE: Egyptian Medical Licensing Exam

	EMLE Prerequisites			EMLE Fees^#^			EMLE Renewal and Validity^#^			
	Portfolio	Reference Letter	Clinical Course	Paid each setting	Free first setting	Paid for promotion	2 years	5 years	Promotion	
Affiliation n (%)										
University	136 (68.7)	53 (26.8)	77 (38.9)	24 (12.8)	137 (72.8)	27 (14.4)	28 (14.7)	85 (44.5)	78 (40.8)	
Ministry of Health	24 (35.5)	10 (16.1)	22 (32.4)	6 (9.5)	38 (60.3)	19 (30.2)	8 (13.1)	27 (44.3)	26 (42.6)	
P-value	<0.001**	0.044*	0.336	0.019*			0.954			
Specialty n (%)										
Academic	46 (59.0)	23 (29.5)	36 (46.2)	14 (18.5)	53 (69.7)	9 (11.8)	12 (15.8)	37 (48.7)	27 (35.5)	
Clinical	108 (61.0)	36 (20.3)	57 (32.2)	16 (9.7)	114 (69.1)	35 (21.2)	21 (12.7)	68 (41.2)	76 (46.1)	
P-value	0.759	0.110	0.033*	0.058			0.305			
^#^^ “^Others” category was excluded from bivariate analysis. *P value ≤ 0.05 **P value <0.001										

The EMLE Admission Time

According to the results of the discussions, some clinical professors stated that “they should finish two years’ medical internship with an accepted portfolio or logbook”. Some academic educators suggested that “The candidate can apply for the EMLE during the second year of internship”.

The survey results added that most of the participants (63%) encouraged allowing the house officers to apply for the EMLE immediately after the end of the two years pre-registration house officers period (internship). The rest of the participants recommended later admission that ranges from two to six months after the completion of the undergraduate studies as shown in Table 2.

Fees and Validity of the License

Most of the participants in the survey (65.8%) proposed that the first-time application for EMLE should be free of charge and that payment should be only in case of the exam retake. Regarding the License validity, 42.1% of participants recommended a 5 years validity period for the license, in addition to completion of Continuous Medical Education (CME) hours during this duration. Another 39.1 % recommend an open validity duration till applying for a new position or promoting, with CME hours during this period. Finally, few participants have recommended 10 years as a validity period or a single life-long licensing as shown in Table 2.

The current study revealed a statistically significant difference between participants’ affiliations and their preferences for EMLE fees, where the majority of participants (72.8%) who work at academic institutes preferred the exams to be paid for, in comparison to participants affiliated to the Ministry of Health who preferred that fees be paid only when exams are related to their promotion, rather than at each setting, even if the first one was waived (p = 0.019).

B. The exam set up

The Exam Settings (the EMLE Test Centers)

Many participants in the online discussion have highlighted the importance of convenient infrastructure, logistics, and human resources. A summary of the participants’ opinions regarding the EMLE test center’s criteria is as follows:

Infrastructure: Responses included the need to have large, equipped, and air-conditioned exam halls, with seating arrangements allowing for distancing, as well as enough measures for exam security. In the case of electronic exams, the responses showed the need for upgraded functioning computers, with high internet connection that is fast and secure. Besides, IT support teams could also include medical educators or e-learning specialists. If OSCE Exams are to be implemented, exam centers should have skill labs, highly simulated mannequins and equipment.

Human resources: Some responses stressed on the importance of having invigilators, in addition to a medical support team to attend to urgent matters, if needed.

Availability and accessibility: Exam centers should be available in terms of number or geographical distribution with the capacity to accommodate a large number of students.

Comfort, safety and ease: This was highlighted in terms of providing elements that allow students to perform without being exposed to internal or external factors, including environmental factors, in terms of seat comfort, adjusted temperatures, proper lighting, aeration and ventilation and noise levels.

Exam instructions: Instructions are to be stated clearly. Instructional notes and posters could be available to aid and remind examinees about exam process, and regulations. Exam support team should be ready and available to respond to inquiries.

The Exam Structure (Blueprint, Methods and Post-Exam Analysis) 

The online discussion participants have reached a consensus regarding the proportional weights of disciplines to be included in EMLE blueprint. They suggested: Internal Medicine (25-30%), Surgery (20-30%), Obstetrics and Gynecology (15-25%) and Pediatrics (15-25%). A considerable number of participants added Primary health care, ICU and Basic Sciences.

The survey participants have added the type of assessment methods and the appropriate number of test items in the exam as shown in Table 4. Most of the respondents (225; 84.6%) recommended to carry-out the EMLE theoretical part in a computer-based setting. 75.2% recommended the use of multiple choice questions (MCQs), extended matching questions (EMQs), and objective structured clinical examinations (OSCEs).

**Table 4:** Survey results “EMLE Setup” (N = 266) EMLE: Egyptian Medical Licensing Exam, CME: continuous medical education, MCQs: multiple choice questions, EMQs: extended matching questions, OSCEs: objective structured clinical examinations

Items	n (%)
Assessment	
Theoretical (e.g. MCQs and EMQs)	57 (21.4)
Theoretical and clinical (e.g. MCQs, EMQs, and OSCE)	200 (75.2)
Others	9 (3.4)
Mode of Administration	
Computer-based	225 (84.6)
Paper-based	41 (15.4)
Number of questions in theoretical components	
100-200 Questions	115 (43.2)
150-300 Questions	101 (38.0)
200-400+Questions	41 (15.4)
Others	9 (3.4)
Duration	
Single session	37 (13.9)
Two sessions within the same day	137 (51.5)
Two sessions on two separate days	85 (32.0)
Others	7 (2.6)
Pass grade	
>50%	34 (12.8)
>60%	177 (66.5)
>70	45 (16.9)
Others	10 (3.8)
Mandatory Duration prior to EMLE Retake	
Immediately after latest EMLE trial	71 (26.7)
3 months or more	148 (55.6)
6 months or more	45 (16.9)
9 months or more	2 (.8)
Others	
Prerequisites for retake	
None	155 (58.3)
CME ± clinical rotation	106 (39.8)
Others	5 (1.9)

Moreover, all the study participants agreed that the exam shall focus on interpretation and case-based scenarios. As mentioned by a clinical educator “The MCQs should be constructed to assess higher cognitive levels and should be based on clinical vignettes to increase clinical reasoning ability for reaching diagnosis, investigation and management options.”

The results also showed that 51.5% of participants recommended the exam to be carried out in two sessions separated by a break on the same day, with every session lasting for 1.5 hours, while 32% recommended to carry out the exam on two sessions also, but on two separate days, each is for two hours for theoretical and another two hours for the clinical assessment sessions as shown in Table 4. Most of the study participants preferred the implementation of two types of assessment methods, including paper and pencil and performance assessment. There was a statistical significance difference between the participants who are academics (87.1%) and participants who work in Ministry of Health as shown in Table 5 (p = 0.014 and 0.023 respectively).

**Table 5:** “EMLE Setup” by Participants’ Affiliation and Specialty EMLE: Egyptian Medical Licensing Exam, CME: continuous medical education

	Assessment Parts^#^		Number of questions in Theoretical Part^#^			EMLE Reset perquisites^#^	
	Theoretical	Theoretical and Clinical	100-200 Questions	150-300 Questions	200-400+ Questions	None	CME ± Clinical rotation
Affiliation n (%)							
University	36 (18.6)	158 (81.4)	72 (37.3)	85 (44.0)	36 (18.7)	107 (55.2)	87 (44.8)
Ministry of Health	21 (33.3)	42 (66.7)	43 (67.2)	16 (25.0)	5 (7.8)	48 (71.6)	19 (28.4)
P-value	0.014*		<0.001**			0.018*	
Specialty n (%)							
Academic	10 (12.8)	68 (87.2)	33 (43.4)	33 (43.4)	10 (13.2)	38 (49.4)	39 (50.6)
Clinical	43 (25.6)	125 (74.4)	79 (46.2)	63 (36.8)	29 (17.0	112 (64.4)	62 (35.6)
P-value	0.023*		0.561			0.025*	
^#^^ “^Others” category was excluded from bivariate analysis. *P value ≤ 0.05 **P value <0.001							

As shown in Table 4, 43.2% of respondents suggested 100-200 test items for the exam, while 38.0% suggested 150-300 items. There was a high statistically significant difference (p <0.001) between the participants’ affiliations and their choice of the number of items; university affiliated participants selected a higher number of questions, in comparison to those who work in the Ministry of Health, as shown in Table 5.

Post-test analysis and psychometrics of the exam were discussed by the participants of the online discussion which favored the selection of MCQs and emphasized on applying the utility formula of assessment, as well as analyzing exam psychometrics.

The Exam Standard-Setting and Pass Mark

Regarding the pass mark of EMLE, most of the participants of the survey 177 participants (66.5%) recommended a score of ≥60% to pass the exam. Forty-five participants (16.9%) recommended the passing score to be ≥70%, while 34 participants (12.8%) suggested the passing score to ≥50% as shown in Table 4. These suggested pass score allocated to the theoretical part and the clinical part separately.

The Exam Retake Policy and Requirements

The survey respondents recommended a time interval for applying for the retake exam and additional prerequisites to be attached as evidence of competencies enhancement. The time interval ranged from immediate retake to a maximum nine months. Most of the participants (155; 58.3%) suggested no added prerequisites before the retake exam, while some participants (106; 39.8%) requested additional prerequisites as interviews and CME hours as shown in Table 4. As shown in Table 5, requirements for retake exams showed statistically significant difference in relation to affiliations.

## Discussion

The current study is important as it suggests a framework for the development of licensing exams that can provide evidence about potential healthcare providers’ competency and determine those who are able to satisfy licensing requirements to practice according to regulatory standards. This would reassure the public that healthcare would be maintained with high-performance providers across the different delivery indicators.

The suggested time for EMLE administration in this study ranged between immediately after, to six months interval after the completion of the two years internship training. In some European countries, like Belgium and Hungary, medical graduates are allowed for registering for licensing exams immediately after obtaining a medical degree, while other countries like Malta require proof of professional practice [16]. Regionally in Saudi Arabia, candidates can apply for the national licensing exam starting from their last year of medical studies. As for medical graduates who obtained their bachelor degree from another country, they can apply if their degrees is recognized by the Ministry of Education [17].

The current situation of licensing in Egypt is based on two obligatory criteria, completion of a bachelor degree in medicine and surgery from an Egyptian university or from an international university that is accredited by the Egyptian Supreme Council of Universities, and completion of the medical pre-registration house officer year(s). The current study suggested other prerequisites for licensing, such as Basic Life Support (BLS) courses, basic computer skills certificate, and an English language certificate. The main purpose of collecting more evidence on skills acquisition is to improve the professional life of the graduates through gaining skills that are essential for their career. The English language plays an important role in medical studies to help graduates to be eligible for international mobility. In line with this concept, Kovacs et al. raised a concern about the effect of language incompetency on patient safety and requested to balance freedom of movement between countries/workplace with language competence. Moreover, basic computer skills are beneficial for reporting and documentation, communication, research, and learning of graduates [17]. After the COVID-19 pandemic, the use of computers and different forms of technology has become mandatory. It is the appropriate technology that would help sustain the medical care services during any crisis [18].

The study highlighted the importance of using a portfolio as a prerequisite for EMLE. Portfolios are beneficial for monitoring and documenting progress in a longitudinal and continuous manner, as well as helping in the development of the physician through reflection and self-assessment [19]. Portfolios will also help the assessors to get a complete picture of the candidates’ clinical performance. The prerequisites for licensing exams vary worldwide. In Saudi Arabia, candidates can apply for the Saudi Medical Licensing Exams (SMLE), after completing the bachelor’s degree in medicine and beginning the internship year training. No further prerequisites are needed [16]. In this study, university-affiliated participants were more in favor of making portfolios a pre-requisite in comparison to their peers at the Ministry of Health. This could be justified by the fact that portfolios are more used as an assessment method in universities than for health practitioners in the Ministry of Health [20].

EMLE is a high-stakes exam; it may shape and control the physician's career. Therefore, it is important for the exam to fulfill all the criteria of objectivity and fairness. The framework suggested in this study included several parameters to ensure these points. First, a test blueprint is an important starting step to be planned in accordance with the National Academic Reference Standards (NARS) and the national competencies of the Egyptian medical graduates. The purpose of the blueprint is to ensure that both content and face validity of the test are determined [21]. Second, triangulation of assessment methods to ensure a wide coverage of the different learning domains. Most of the participants suggested that the MCQ will be a sufficient assessment tool for the first part of the exam. Although its preparation is time-consuming and needs well-trained staff to develop, MCQs will ensure objectivity, a wider sample, easier scoring, and shorter time for answering and a feasible format for reliable automated grading. However, its preparation is time-consuming and needs a well-trained staff to develop [22].

It is important to bear in mind that the exam is multidisciplinary and should be testing different levels and domains. Therefore, The MCQ format may be more applicable as it can be used in large numbers. These advantages guided the recommendation of the test item number in our study that ranged from 100-200 items to 150-300 MCQs. It is also important for MCQs to be constructed to assess higher cognitive levels, such as analysis, synthesis, and evaluation, and be based on clinical vignettes to increase the likelihood of integrating the clinical sciences (e.g., diagnosis, investigation and management options). In the United States, the first part of USMLE consists of a 322 MCQ to measure the basic science knowledge, divided into seven blocks with 46 items per block, and each block for one hour with a maximum of seven hours testing [23].

Furthermore, other types of summative assessments were suggested; clinically related Short Answer Questions (SAQs), and Modified Essay Questions (MEQs) to assess theoretical knowledge and OSCE and simulated clinical exams were the best choices for the clinical skills assessment. This is the current situation in many countries all over the world like Canada, Ireland, Korea, Switzerland, the United States, and the United Kingdom [2]. In Saudi Arabia, for the cognitive domain, they use MCQs or SAQs test formats and OSCE with a standardized patient or observed short case for assessing both Psychomotor and Affective domains [24]. The OSCE will provide a valid, standardized, and reliable clinical exam. It is the golden exam in most of the postgraduate degrees [25,26].

Adding another important point to the fairness and objectivity of the exam, most participants recommended a standard set of ≥60% to pass each part of the exam separately. Additionally, the post-exam psychometrics analysis is of high importance as it considers the quality check of the exam items after its implementation. For instance, the Difficulty Index could gauge how 'hard' or 'easy' the question is [27]. In addition, there is the Discrimination Index, where questions distinguish between higher and lower performing students [28]. Finally, there is the Distractor Effectiveness (DE), an indicator of alternatives' ability to distract from the correct answer [29].

It is important to consider that the NLE is an obligatory exam. Therefore, flexibility and practicality are essential. This can be reflected in the retake policy, fees, and license validity period. The retake policy is recommended to be flexible in terms of time and requirements. In Saudi Arabia, all eligible candidates have the chance for three exam sittings since their first attempt if they failed to achieve a passing score [16].

Regarding the fees, the current study defends the support of the medical graduates by waving them from the exam fees for the first time. It is an essential issue to be considered that the country provides the exam for the first attempt with free or even at low cost in relevance to the salary provided to the candidate during their internship training year. Exam costs should be considered, as they differ among different countries due to the nature of the implemented exams, available resources, and number of applicants. In the United States, licensing exam total fees would amount to 3200 USD, comparable to 800 USD paid by international medical graduates [2]. While the absolute amount is higher in the case of the United States, the corresponding amount is much higher for international medical graduates, including Egyptian medical graduates, in comparison with individual and gross national incomes [23]. Therefore, the salaries of the medical graduates should be considered [30].

Finally, the validity of the license was a point of difference and debate between the participants. The validity period may vary but the fact that the NLE is a discriminating exam should be considered. Accordingly, it may be better to give the applicants the time for learning and maturity before re-taking the exam. Moreover, taking the exam several times may create wise students who can beat the system and pass the exam easily. Adding to this, the burden that will be added on the test developers’ team for continuous development, revision and adding new test items to safeguard the test from losing its main purpose. The current study revealed no differences between clinicians and academics in respect to their responses towards the validity of the license. More in both categories chose open validity until promotion. Promotion is a valued merit where progress in the field, acknowledgement by peers and salaries may depend upon, yet it was the least option that required renewal. Regular intervals of certification and providing CME proof are universally recognized as means of ensuring quality of profession and updating knowledge and competencies [30].

Overall, the study showed the perceptions and aspirations of the medical education community towards the EMLE and enabled the development of a framework of EMLE that could guide its planning, implementation and evaluation.

## Conclusions

The current study has developed a framework for the Egyptian National Medical Exam. The framework focused on two main themes: the exam logistics and the exam setting. A multidisciplinary team was suggested for the exam committee together with convenient infrastructure, logistics, and human resources for the exam setting. Fairness and objectivity were highlighted through several factors: development of exam blueprint, types of the assessment methods and post-exam analysis. Finally, the retake policy, fees and validity of the license was recommended with a student-centered perspective. We tried to suggest a roadmap through a critical reflection for the best of the Egyptian health care system. We want to stress that this exam is not "the Magic Solution" for all our medical education system’s challenges in Egypt. But it is considered as a good starting point towards standardization and globalization of the Egyptian medical education. Additionally, it is a recommendation for further study to explore the views of the students about the EMLE after its real implementation by the year 2023.

Although the current study used the suggestions of medical practitioners and educators regarding the suggested framework of the EMLE development, the results might have related to the experiences of the presented sample and therefore not be generalizable to a larger population. Despite this, we have tried to use all the available platforms, multiple mixed methods and reach diverse participants.

## Appendices

Appendices

Appendix 1 - Online Listserv Discussion

Topic: Egyptian Medical Licensing Exams (EMLE) for 2023 Medical Graduates

Objectives

1)    Justify the need for establishment of licensing exams for the Egyptian Medical graduates.

2)    Establish the criteria of selection of multidisciplinary team who will responsible for EMLE construction.

3)    Establish EMLE admission criteria for the Egyptian graduates.

4)    Discuss the EMLE blueprinting construction.

5)    Select other appropriate method of summative assessment for EMLE.

6)    Discuss methods of MCQs post exam analysis.

7)    Discuss the criteria needed for an exam center to be able to conduct the EMLE.

Question (1) for 1 day

            The drive towards standardization in medical regulation can be understood as a response to the internationalization of medical training and, more generally, the increasing mobility of the medical workforce in a globalized world. The Egyptian medical regulators announced that it is planned to establish a national licensing examinations in 2023 it laid out a timeframe for the introduction of a Medical Licensing exams which will ultimately be taken by all graduate for the New Egyptian Medical Curriculum (5+2)

            Today we need to start our discussion with this important question, why there is a need for establishment of licensing exams for the medical graduates of the New Egyptian Medical Curriculum (5+2)?

Question (2) for 2 days

Egypt is going to have a national license exam for medical graduates to ensure that graduates are safe doctors who are fit to practice medicine, a passionate professional multidisciplinary team should gather for the planning and conduction of the exam. Putting these goals in front of our eyes the authority responsible for the exam should choose the examiners precisely.

From your point of view, from which disciplines the multidisciplinary team will be constructed and what is the criteria of selection of this team?                

Question (3) for 2 days

The graduates of the new Egyptian Medical curriculum (5+2) will have an internship duration for two years which give them enough time for preparation to this medical licensing exam so we need to determine certain points and criteria for admission of the Egyptian National license exam.

A.      From your point of view, what is your suggested plan for the internship -2 years’ duration- in 5+2 curriculum?    (day 1)  

B.    What is/are the perquisite admission criteria needed before entering this licensing exams for the medical graduates of the New Egyptian Medical Curriculum (5+2)?    (day 2)

Question (4) for 2 days

You are a member among the committee responsible for the Egyptian National Licensing Examination which will be carried out in 2023 and you reached a decision to use MCQs for assessment of the basic and clinical knowledge. Being one of multidisciplinary team members who will share in the preparation of the EMLE try to help the committee in the below important points:

A.    Put the layout of subjects’ weight % included in MCQ blueprint of the EMLE?

     What is the total number of MCQs will be used in EMLE and duration needed for this exam?

B.    From your point of view, what other methods of summative assessment is needed to be included beside MCQ? (Day 2)

Question (5) for 2 days

Being a member of Exam Revision Committee - a Task Force subcommittee who are assigned to review the contents and analyze the results of MCQs of the EMLE.

A.    What are Psychometrics of MCQs that you should consider while preparing the exam? (Day 1)

B.    While analyzing 2 cycles of Egyptian Licensing Exams of medical graduates, you noticed that some questions are usually answered by few candidates.

         Is there any measurement way to describe an item for being difficult and how can we analyze the MCQ items of the EMLE?

         What is your management plan based on these item analysis findings?

Question (6) for 2 days

The Egyptian Medical Licensing Exam (EMLE) committee has appointed different members to act as an advisory group. You were appointed due to your expertise.  The committee suggests that the EMLE has to be carried out in multiple exam centers in different governorates due to the large number of students across Egypt. Each center would have to fulfill specific standards in order to be approved by the EMLE committee as an exam center. The advisory group should make sure that the selected exam centers fulfil these criteria, noting that the current proposed mode is an MCQ test to assess students’ medical knowledge.

A.    In your opinion, what are the minimal criteria/standards that should be available in these centers thus ensuring a fair and confidential test, and the comfort of the examinees, and their supervisors? You could discuss at least three criteria and clarify the reasons behind your selection.

B.    If the MCQ exam is computer based rather than being a paper test, would you consider a change in the criteria you mentioned?

C.    If the EMLE would consider expanding their assessment to include an OSCE component to assess the examinees’ clinical, professional and ethical competencies. In your opinion, what are the additional requirements needed for exam centers to accommodate adding an OSCE component to the EMLE?  (Day 2)

Last day: Wrap up of the discussion.

Appendix 2 - Online Survey Questions

Egyptian Health Care Professions` Perceptions of the National Licensing Exam

I.               Demographic data

Are you working in the Ministry of Health or a university staff member?

-          Ministry of Health

-          University member

-          Other

Faculty:

University (Only if you are a university staff member):

Specialty:

Professional rank:

·       Demonstrator

·       Assistant Lecturer (university staff member)

·       Specialist (not a university staff member)

·       Lecturer (university staff member)

·       Consultant (not a university staff member)

·       Associate/assistant professor

·       Professor

II.            Admission Criteria for Egyptian Medical Licensing Exam (EMLE)

Select the proposed time in the internship 2 year program that allow the house officer to apply for the EMLE

-          Immediately after internship 2 years’ program

-          First 2 months after internship 2 years’ program

-          3 Months after internship 2 years’ program

-          More than 4 months

-          Other please specify

Besides completing the MBBS degree and 2 internship years, please select all the obligatory criteria for admission of the exam that you recommend

-          An English language certificate passing a minimum determined score

-          A basic computer skills certificate (eg ICDL)

-          Basic life support (BLS) certificate

-          Finishing a research project

-          Accepted internship portfolio /logbook

-          Recommendation letter/s from internship supervisor/s

-          Certain number of conferences attendance

-          CME proof for soft skills as leadership, communication skills

-          CME proof for publication

-          CME proof for medical ethics

-          Passing a formative assessment simulating the licensing exam during the internship with a certain score

-          Attending a training course on EMLE questions

For the previous question, do you have any other recommendations for obligatory admission criteria for EMLE

III.          EMLE Structure

In your opinion, what are the reasonable assessment tools for EMLE?

-          MCQs only

-          MCQS & Extended matching

-          MCQS & OSCE

-          MCQs, MEQs and OSCE

-          Other requirements (Please write your suggestion it in the next  question box)

In your opinion, how many questions should be included in the theoretical exam?

-          100-200

-          150-300

-          200 - 400

-          More than 400

-          Others (Specify)

EMLE should be conducted by which of the following mode?

-          Computer based exam

-          Paper format

In your opinion, what is the suggested allocated time for EMLE?

-          One session for 2 hours

-          2 sessions separated by break on the same day (every session one and half hour)

-          2 sessions on 2 separate days (2 hours for theoretical and another session for clinical assessment)

-          Others (Specify)

What is the suggested standard setting for the exam that the candidate should achieve to pass?

-          Above 50%

-          Above 60%

-          Above 70%

-          Others (specify)

IV.          EMLE Reset Rules

In your opinion and in case of those candidates failed to success on the first exam trial, what is the time interval that the candidate should spend before applying for reset?

-          Apply immediately

-          At least 3 months interval

-          At least 6 months interval

-          At least 9 months interval

In your opinion, do you think there should be displayed prerequisites that should be full-filled before being eligible to do the reset?

-          No requisites

-          CME hours

-          CME hours and more clinical training rotations

-          Others (Specify)

In your opinion and among the following, what is the best option for payment fees of EMLE?

-          Paid for the first time and for each reset

-          Free for the first time and paid only on reset

-          Paid only on upgrading

-          Others (Specify)

After obtaining the license to practice medicine, what is the suggested time for license renewal (expiry date)?

-          2 years + CME hours.

-          5 Years + CME hours.

-          Only when get new certificate (upgrading) + CME hours

-          Others (Specify)

## References

[1] Archer J, Lynn N, Coombes L, Roberts M, Gale T, de Bere SR (2017). The medical licensing examination debate. Regul Gov.

[2] Price T, Lynn N, Coombes L, Roberts M, Gale T, de Bere SR, Archer J (2018). The international landscape of medical licensing examinations: a typology derived from a systematic review. Int J Health Policy Manag.

[3] Swanson DB, Roberts TE (2016). Trends in national licensing examinations in medicine. Med Educ.

[4] Borow M, Levi B, Glekin M (2013). Regulatory tasks of national medical associations - international comparison and the Israeli case. Isr J Health Policy Res.

[5] Shams T, El-Masry R, Al Wadani H, Amr M (2013). Assessment of current undergraduate anesthesia course in a Saudi University. Saudi J Anaesth.

[6] Abdelaziz A, Kassab SE, Abdelnasser A, Hosny S (2018). Medical education in Egypt: Historical background, current status, and challenges. Health Professions Education.

[7] Schomaker R (2015). Accreditation and quality assurance in the Egyptian higher education system. Qual Assur Educ.

[8] Aboshady OA, Radwan AE, Eltaweel AR (2015). Perception and use of massive open online courses among medical students in a developing country: multicentre cross-sectional study. BMJ Open.

[9] Gukas ID (2007). Global paradigm shift in medical education: issues of concern for Africa. Med Teach.

[10] Loveluck Loveluck, L. (2012 (2020). Education in Egypt: Key Challenges. https://d1wqtxts1xzle7.cloudfront.net/31814727/0312egyptedu_background.pdf?1378024050=&response-content-disposition=inline%3B+filename%3DThe_views_expressed_in_this_document_are.pdf&Expires=1619116709&Signature=Wz-DkgB55DLSBSc89Q9ywsRdwAHVr1jUXuI1-4s5Ze89bez8-BCW0iB8v7hmTivZOkQxCHOBNAHNSs21keEd5QjwCXiVZILSxO7hjbP6nDhpwTL4h9sEfgZ0LNVL9iGQnE3xpwslh~KjNTvWLawLV6L6bkoJhz2~W4aws2-znsy4CN3WaQ6wen0amKxGgL3uRymDz9bFJZUQVHP0zwrut2q~k0vqPSFkPCKbvlM10tCdVl3Fc2OQhl0nK5S7vC--BNF3lGlXv6ZEDfBeeiwvmLTbgNkugo8vpQG8jNC8KPkU765t1LNL9Ws9uh~ceZRhaXiEhE5RKosOpzS-eBl1zA__&Key-Pair-Id=APKAJLOHF5GGSLRBV4ZA.

[11] Tekian A, Boulet J (2015). A longitudinal study of the characteristics and performances of medical students and graduates from the Arab countries. BMC Med Educ.

[12] van der Vleuten CP (2009). National, European licensing examinations or none at all?. Med Teach.

[13] (2020). WFME Recognition Programme. https://wfme.org/accreditation/recognition-programme.

[14] (2018). Egyptian bylaws and regulation for university regulation in MBBCh degree, Decree No. 565 of 2018. Decree No. 565 of.

[15] (2020). World Medical Association Declaration of Helsinki: Ethical Principles for Medical Research Involving Human Subjects. https://www.wma.net/policies-post/wma-declaration-of-helsinki-ethical-principles-for-medical-research-involving-human-subjects/.

[16] Kovacs E, Schmidt AE, Szocska G, Busse R, McKee M, Legido-Quigley H (2014). Licensing procedures and registration of medical doctors in the European Union. Clin Med (Lond).

[17] (2020). General Assessment Bylaws. https://www.scfhs.org.sa/en/examinations/Regulations/General%20Assessment%20Bylaws.pdf.

[18] Ward JP, Gordon J, Field MJ, Lehmann HP (2001). Communication and information technology in medical education. Lancet.

[19] Abouzeid E, Abdel Nasser A (2018). Evaluation of the portfolio’s implementation in clinical clerkship: students’ and staff’s perception in Egypt. J Med Educ.

[20] Heeneman S, Driessen EW (2017). The use of a portfolio in postgraduate medical education - reflect, assess and account, one for each or all in one?. GMS J Med Educ.

[21] Raymond MR, Grande JP (2019). A practical guide to test blueprinting. Med Teach.

[22] Norcini J, Anderson B, Bollela V (2011). Criteria for good assessment: consensus statement and recommendations from the Ottawa 2010 Conference. Med Teach.

[23] McMahon GT, Tallia AF (2010). Perspective: anticipating the challenges of reforming the United States medical licensing examination. Acad Med.

[24] Bajammal S, Zaini R, Abuznadah W (2008). The need for national medical licensing examination in Saudi Arabia. BMC Med Educ.

[25] Fouad S, Gouda E, Abdel Nasser A, Kamal D (2019). Perception of students, staff and simulated patients towards objective structured clinical examination (OSCE). Educ Med J.

[26] De Swardt M, Jenkins LS, Von Pressentin KB, Mash R (2019). Implementing and evaluating an e-portfolio for postgraduate family medicine training in the Western Cape, South Africa. BMC Med Educ.

[27] El-Uri FI, Malas N (2013). Analysis of use of a single best answer format in an undergraduate medical examination. Qatar Med J.

[28] Mukherjee P, Lahiri SK (2015). Analysis of multiple choice questions (MCQs): item and test statistics from an assessment in a medical college of Kolkata, West Bengal. IOSR J Dent Med Sci.

[29] Rao C, Kishan Prasad HL, Sajitha K (2016). Item analysis of multiple choice questions: assessing an assessment tool in medical students. Int J Educ Psychol Res.

[30] Kamal Elden NM, Ibrahim Rizk HI, Wahby G (2016). Improving health system in Egypt: perspectives of physicians. Egypt J Comm Med.

